# Psychometric and clinical evaluation of schizophrenia remission criteria in outpatients with psychotic disorders

**DOI:** 10.1186/s12888-023-04701-3

**Published:** 2023-03-28

**Authors:** Karolina Sakinyte, Christopher Holmberg

**Affiliations:** 1 Department of Psychotic Disorders, Sahlgrenska University Hospital, Gothenburg, Sweden; 2 Institute of Health and Care Sciences, University of Gothenburg, Gothenburg, Sweden

**Keywords:** Factor analysis, Outcome measure, Psychotic disorder, Remission, Schizophrenia, Validation study

## Abstract

**Background:**

Psychotic disorders such as schizophrenia have debilitating effects on health and functioning. Given symptomatic remission’s recent emergence as a viable treatment goal, the Remission in Schizophrenia Working Group’s criteria (RSWG-cr), based on eight items from the Positive and Negative Syndrome Scale (PANSS-8), are frequently used in clinical and research settings. Against that background, we sought to evaluate the PANSS-8’s psychometric properties and examine the RSWG-cr’s clinical validity among outpatients in Sweden.

**Methods:**

Cross-sectional register data were collected from outpatient psychosis clinics in Gothenburg, Sweden. Following confirmatory and exploratory factor analyses of PANSS-8 data (*n* = 1,744) to assess the PANSS-8’s psychometric properties, internal reliability was evaluated using Cronbach’s alpha. Next, 649 of the patients were classified according to the RSWG-cr and their clinical and demographic characteristics compared. Binary logistic regression was used to estimate odds ratios (OR) and assess each variable’s impact on remission status.

**Results:**

The PANSS-8 showed good reliability (α = .85), and the 3D model of psychoticism, disorganization, and negative symptoms presented the best model fit. According to the RSWG-cr, 55% of the 649 patients were in remission; they were also more likely to live independently, be employed, not smoke, not take antipsychotics, and have recently received a health interview and physical examination. Patients living independently (OR = 1.98), who were employed (OR = 1.89), who were obese (OR = 1.61), and who had recently received a physical examination (OR = 1.56) also had an increased likelihood of remission.

**Conclusions:**

The PANSS-8 is internally reliable, and, according to the RSWG-cr, remission is associated with variables of interest for patients’ recovery, including living independently and being employed. Although our findings from a large, heterogeneous sample of outpatients reflect everyday clinical practice and reinforce past observations, the directions of those relationships need to be assessed in longitudinal studies.

**Supplementary Information:**

The online version contains supplementary material available at 10.1186/s12888-023-04701-3.

## Introduction

Worldwide, psychotic disorders (e.g., schizophrenia), with core symptoms including hallucinations, delusions, disorganized thought and speech, avolition, and lack of emotional expression [1], represent some of the most debilitating psychiatric conditions [2]. Moreover, their high rates of cardiometabolic and psychiatric comorbidities, as well as cognitive and functional impairments [3], can significantly decrease life expectancy compared with the general population [4]. Given the absence of objective methods of diagnosing psychotic disorders, clinical observation using symptom rating scales is common [5, 6]. For better functional recovery, the psychopharmacological treatment of such disorders chiefly targets the remission of symptoms [7, 8].

### Definition of *remission* in psychotic disorders

Early descriptions of schizophrenia characterized it as a progressive disease with little hope of recovery [9]. However, that pessimistic view on the prognosis has eroded in recent decades as remission and recovery have emerged as potentially viable treatment goals [9, 10]. Nevertheless, insight into those concepts has been limited by inconsistent definitions of *remission* across studies [11].

In 2005, the Remission in Schizophrenia Working Group (RSWG) published consensus criteria for remission (RSWG-cr) to be used in clinical settings and research [12]. Representing two components—symptom severity and time—the RSWG-cr use core items from established scales including the Positive and Negative Syndrome Scale (PANSS), a widely used clinician-rated scale with 30 items divided into three categories—positive symptoms, negative symptoms, and general psychopathology—and rated on a 7-point scale from 1 (*absent*) to 7 (*extreme*) [13]. For symptom severity, the RSWG-cr provides a score of ≤ 3 points corresponding to mild severity or less on the eight items of the PANSS-8: P1 (delusions), P2 (conceptual disorganization), P3 (hallucinatory behavior), N1 (blunted affect), N4 (social withdrawal), N6 (lack of spontaneity), G5 (mannerisms and posturing), and G9 (unusual thought content). Those items are assumed to map onto three dimensions of psychopathology: psychoticism (P1, P3, and G9), disorganization (P2 and G5), and negative symptoms (N1, N4, and N6). Meanwhile, for the component of time, the RSWG-cr posits that, to indicate remission, the criteria for symptom severity need to be met for at least 6 months.

Since their publication, the RSWG-cr have been widely employed to define *remission* in psychotic disorders [14]. In Sweden, a longitudinal validation study revealed that remission according to the RSWG-cr was usually stable over time, that all PANSS-8 items except G5 efficiently help to distinguish remitted from non-remitted patients, and that the contribution of the remaining PANSS items is not substantial enough to warrant inclusion in the RSWG-cr [15]. A recent meta-analysis of longitudinal studies of first-episode psychosis revealed 52% remission frequency over 4 years when using RSWG-cr, with considerable heterogeneity between studies [16]. However, sociodemographic and clinical predictors did not significantly affect remission [16].

Symptomatic remission is arguably a necessary step toward broader recovery, including improved psychosocial functioning and well-being [10]. Though lacking any standard definition, functional recovery is usually a central treatment goal in schizophrenia [17–19]. Nevertheless, a recent review has highlighted employment status as a key outcome in functional recovery [20], and being employed has been associated with higher health-related quality of life among patients with schizophrenia [21].

While remission according to RSWG-cr has been found to predict increased occupational functioning, social functioning, and quality of life [22–25], other research has shown only a slight difference in daily functioning [26]. More stringent criteria as predictors of sustained remission over time [15, 27] and functional improvement [28] have thus been advocated. Nevertheless, the RSWG-cr are frequently used, widely recognized as being clinically valuable [15, 22], and recommended for assessing remission per clinical guidelines in Sweden [29].

### Objectives

In our study, we aimed to evaluate the PANSS-8’s psychometric properties and explore the RSWG-cr’s clinical validity in a naturalistic sample of Swedish outpatients with psychotic disorders. We thus defined two objectives:

1. To evaluate the PANSS-8’s psychometric properties in factor analyses; and
2. To assess the RSWG-cr by comparing the sociodemographic and clinical characteristics of patients in remission versus not in remission.

## Methods

### Design

Our study followed an observational, cross-sectional study design.

### Data collection and sampling

We used data from the local patient registry in the Department of Psychotic Disorders at Sahlgrenska University Hospital in Gothenburg, Sweden [30]. The data were collected at the outpatient clinics from 2016 to 2019 during annual checkups offered to all patients registered at the clinics, including ones with schizophrenia, schizoaffective disorder, and other psychotic disorders. The specific diagnoses given to individual patients were not registered. The checkups, administered to monitor patients’ health status, generally gather sociodemographic and clinical background data and responses on rating scales, including the PANSS-8; the protocol that clinicians use when recording data appears in Supplementary File 1. During the checkups, patients are also offered physical examinations and health interviews. Because each patient in the registry may have multiple entries, only the first entry between 2016 and 2019 was included for patients with several entries. Before analysis, data were anonymized to avoid disclosing patients’ identities.

**Fig. 1:**
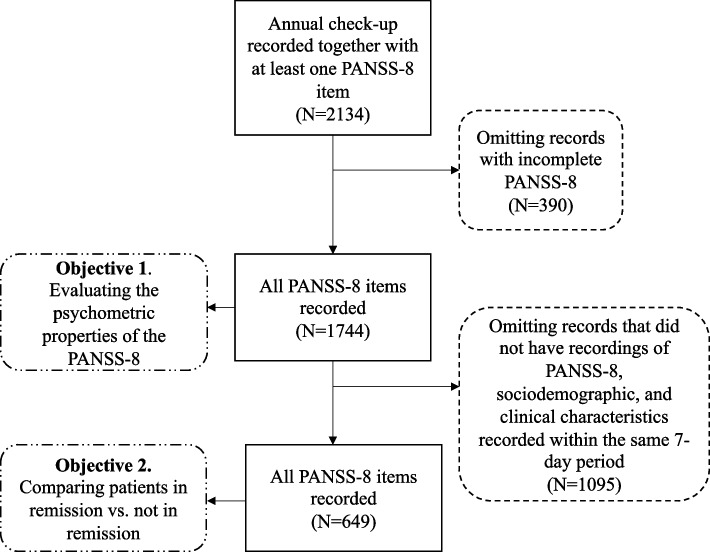
Sampling procedure

For Objective 1—the psychometric evaluation of the PANSS-8—all patients with registered ratings for all items on the scale were included (Fig. 1). For Objective 2, a different data set with more clinical characteristics was examined. Data in analyses for Objective 2 needed to be registered within the same 7-d period to allow meaningful analysis and associations. Given the need to include concurrent patient data while still requiring the registration of all eight PANSS-8 items, fewer patients were included in those analyses than in analyses for Objective 1 (Fig. 1). In being relatively small, the sample was deemed inappropriate for the psychometric analyses for Objective 1.

### Variables

Remission, used as the grouping variable for between-group comparisons and as the dependent variable in logistic regression analyses, was defined as a score of 3 (mild) or less on all eight items, corresponding to RSWG-cr’s symptom severity component [12]. The PANSS-8 sum score, constructed to reflect “overall symptom severity” and allow certain parametric tests to be conducted, was calculated by adding the ratings for each item. The time component, however, could not be applied given the data’s cross-sectional nature.

Age in years and sex (*male* or *female*) were recorded for all patients in the samples for both objectives. The sample for Objective 2 also included other sociodemographic and clinical variables possibly relevant to the progression and outcome of psychotic disorders, as suggested by research and clinical experience [3, 4, 8, 9, 18, 20, 24, 25, 31–38]. Meanwhile, categorical variables were dichotomized to obtain sufficiently large groups to allow statistically robust comparisons.

Sociodemographic variables were place of birth (*Sweden* vs. *Outside Sweden*), level of education (*High school not completed* vs. *High school completed*), type of income (*Personal income* vs. *Social benefits)*, and living situation (*Independent living* vs. *Assisted living*). We examined those sociodemographic variables because migration is a known risk factor for schizophrenia [32]; individuals with schizophrenia experience a wide range of psychosocial difficulties [39]; and unemployment among people with schizophrenia is high, likely due to extrinsic factors (e.g., discrimination and stigma) and their cognitive and social difficulties [33].

Among the clinical variables were duration of diagnosis, meaning the year when the psychotic disorder was diagnosed (*2009 or earlier* vs. *2010 or later*), with a cutoff chosen because diagnoses for some years before 2010 were unavailable, and BMI (Body Mass Index; kg/m^2^). Six other clinical variables were recorded as *Yes* or *No*: smoking status, obesity (BMI ≥ 30), specified somatic comorbidities, current treatment with antipsychotic medication, health interview registered in the past year, and physical examination registered in the past year. We chose to examine those variables because, for one, psychosis that has long gone untreated is associated with poor outcomes [36]. For another, smoking cigarettes is relatively prevalent among patients with schizophrenia, which possibly reflects the use of nicotine as self-medication [40, 41]; however, other factors include shared genetic vulnerability, confounded by the use of illicit substances or other social factors, and smoking’s causal effect on the development of psychosis [34]. Somatic comorbidity is also a major concern in psychosis, and many antipsychotic medications (e.g., olanzapine and clozapine) have metabolic side effects, including weight gain [37]. Lifestyle factors such as smoking and physical inactivity, which are relatively prevalent among people with schizophrenia, also contribute to cardiovascular risk [35]. Disease-related factors including cognitive impairment and lack of motivation may reduce such individuals’ propensity to care for their physical health and seek care when needed [38]. Other evidence suggests that patients with schizophrenia receive less treatment for somatic conditions than their mentally healthy counterparts [38, 42].

Last, our clinical variables were the presence (*Yes*) or absence (*No*) of six somatic comorbidities: diabetes mellitus, cardiovascular disease, thyroid disease, kidney disease, chronic obstructive pulmonary disease, and cancer. Those variables were pooled in a variable, somatic comorbidity, indicating the presence of any of them. Although the list of six diseases is not exhaustive, the somatic comorbidity variable is a good indicator of general somatic health status because it captures the leading causes of death in the general population (e.g., cardiovascular disease and cancer) and indicators of poor metabolic health (e.g., diabetes mellitus), which is of central concern for patients with psychotic disorders.

### Analysis

#### Analyses for Objective 1

The mean, standard deviation, and median of the score distributions of each PANSS-8 item were calculated. Floor effects, meaning the percentage of the sample with the lowest-possible score (1 = *absent*), and ceiling effects, meaning the percentage of the sample with the highest-possible score (7 = *extreme*), were calculated for each item and the whole scale.

The PANSS-8’s psychometric properties were initially evaluated using confirmatory factor analyses (CFA), which generally evaluate predefined hypotheses about the number and structure of factors against data [43]. Two predefined models were compared. The first had three factors based on the original PANSS’s subscales: positive symptoms (P1–P3), negative symptoms (N1, N4, and N6), and general psychopathology (G5 and G9) [13]. Meanwhile, the second had three factors corresponding to the historic dimensions of psychopathology proposed by the RSWG [12]: psychoticism (P1, P3, and G9), disorganization (P2 and G5), and negative symptoms (N1, N4, and N6).

Because the data did not show significant tendencies toward non-normality (skewness < 2.00, kurtosis < 7.00), maximum likelihood estimation was used [44]. Relationships between observed data and data expected from the hypothesized models were evaluated by computing the following goodness-of-fit indices, with thresholds of acceptable fit shown in parentheses: comparative fit index (CFI, > 0.90), Tucker–Lewis index (TLI, > 0.90), root mean square error of approximation (RMSEA, < 0.10), and standardized root mean square residual (SRMR, < 0.08) [45]. Normed chi-square (NC), meaning the chi-square value divided by degrees of freedom, was also calculated given its relative insensitivity to the effects of samples exceeding 400 [46], with NC values exceeding 5.00 considered to be acceptable [47]. However, that approach may not properly adjust for exceptionally large samples when the instrument tested contains few items, for such instruments have fewer degrees of freedom.

Convergent validity, meaning correlation between variables expected to be related, and discriminant validity, meaning no correlation between variables expected to be unrelated, was computed by correlating items with their own and other factors using Pearson’s *r* (≥ 0.80 = *strong correlation*, 0.60– 0.79 = *moderate correlation*, < 0.60 = *weak correlation*) [48].

Internal reliability was tested using Cronbach’s alpha for subscales containing three items. Although Cronbach’s alpha values ≥ 0.70 are often considered to indicate acceptable reliability, ≥ 0.8 is arguably a more appropriate cutoff in most contexts [49]. For the subscales General Symptoms and Disorganization, each with only two items, Cronbach’s alphas were supplemented with Spearman–Brown scores, which are arguably more accurate than Cronbach’s alphas when assessing the internal reliability between two items [50].

An exploratory factor analysis (EFA) was conducted to supplement the initial CFA and thereby potentially identify other latent factors. In that case, a principal components analysis was conducted using varimax rotation, and components (i.e., factors) with eigenvalues ≥ 1.0 were retained [51]. A CFA on the resulting EFA model was performed.

#### Analyses for Objective 2

Normality for continuous variables was assessed using P–P and Q–Q plots, supplemented with skewness and kurtosis values, as shown in Supplementary File 2.

To assess the distribution of demographic and clinical characteristics in remitted versus non-remitted patients, between-group comparisons were performed using chi-square tests for categorical variables. For continuous variables, independent sample *t* tests were used for the normally distributed variables (age and BMI), whereas the Mann–Whitney *U* test was used for the non-normally distributed variable (PANSS-8 sum score).

Before logistic regression analysis, bivariate association tests between potential predictors—that is, the sociodemographic and clinical variables in analyses for Objective 2—and the control variables of sex and age were conducted. Pearson’s *r* and Spearman’s rho were used as measures of correlation, with 0.7 as the maximum value for inclusion in the model without an unacceptable risk of multicollinearity. Variance inflation factor and tolerance were also employed as diagnostics of multicollinearity, with threshold values of < 5.0 and > 0.2, respectively [52].

To assess and quantify the independent contributions of demographic and clinical variables as predictors of remission status, a binary logistic regression analysis was performed using the enter method, with remission (*Yes* or *No* according to RSWG-cr) as the dependent variable. The PANSS-8 sum score and BMI were excluded as predictor variables given their inherent relationships with RSWG-cr and obesity, respectively. Because logistic regression models do not account for missing data, only patients with data for all variables were included [53]. Odds ratios (OR) were calculated with confidence intervals (CI) defined at the 95% level.

For Objectives 1 and 2, all statistical analyses were performed in SPSS version 28.0 (IBM), and statistical significance was indicated by two-tailed *p* values of < 0.05.

## Results

### Characteristics of the samples

#### Sample for Objective 1

The sample used to evaluate the PANSS-8’s psychometric properties contained 1,744 patients. Mean age was 52 years (*SD* = 14.0, range: 19–93), and 44% were women. The age and sex distribution did not differ significantly (*p* = 0.06 and *p* = 0.85, respectively) from the smaller sample used for Objective 2. The mean PANSS-8 sum score was 16.4 (*SD* = 7.8, range: 8–51), which was significantly higher than the smaller sample’s (*p* < 0.001).

#### Sample for Objective 2

The sample used to assess the RSWG-cr included 649 patients aged 51 years on average (*SD* = 13.8, range: 19–92). Of them, 45% were women, 63% were born in Sweden, 53% lived independently, 70% had completed high school, and 31% had their own income. While 29% had at least one somatic comorbidity (e.g., diabetes and cardiovascular disease), 23% were smokers. Mean BMI was 29.1 (*SD* = 6.2), and 39% were obese (BMI ≥ 30). While 73% were diagnosed with a psychotic disorder in 2009 or earlier, 91% were currently being treated with antipsychotic medication. In the year preceding data collection, 67% of the patients had a registered health interview and 63% a registered physical examination. The mean PANSS-8 sum score was 15.3 points (*SD* = 7.6, range: 8–42).

### Objective 1: Factor analyses

The distributions of scores on individual PANSS-8 items appear in Supplementary File 3. The mean score was < 3 for all items, and all items showed pronounced floor effects. The largest floor effect, 68%, was for item G5 (mannerisms and posturing), which also had the lowest mean score, 1.59.

**Fig. 2:**
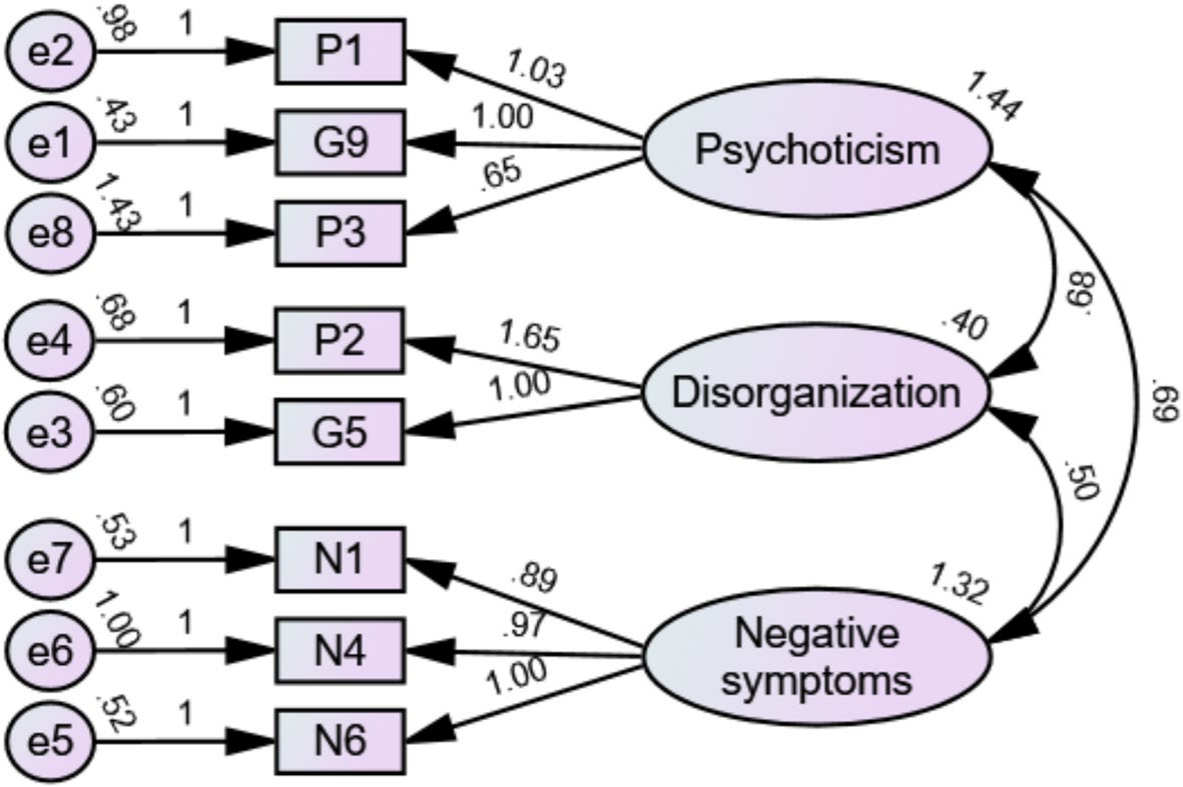
*CFA of the PANSS-8 based on the 3D model proposed by the RSWG (12), with correlations denoted as Pearson’s* r *values. Fit indices: NC* = *13.818, CFI* = *0.967, TLI* = *0.940, SRMR* = *0.044, RMSEA* = *0.086*

CFA was used to compare three models: two predefined models corresponding to the original PANSS subscales (positive, negative, and general symptoms) and the dimensions proposed by the RSWG (psychoticism, disorganization, and negative symptoms) and a two-factor structure generated by EFA (positive/psychotic symptoms and negative symptoms) with item G5 excluded. The details of all the tested models and the EFA appear in Supplementary File 4. The second model showed the best fit indices (Fig. 2), all of which had acceptable values except for NC.

Concerning the three dimensions, Cronbach’s alpha was 0.76 for psychoticism, 0.64 for disorganization (Spearman-Brown value = 0.65), and 0.84 for negative symptoms. Cronbach’s alpha for the full PANSS-8 was 0.85.

### Objective 2: Remitted and non-remitted patients compared

Patients were divided into two groups based on their scores for PANSS-8 items according to the RSWG’s criteria for severity. The remission group consisted of 356 patients (55%) with a rating of ≤ 3 on all items, whereas the remaining 293 patients (45%) who scored ≥ 4 on at least one item formed the non-remission group (Table 1).

**Table 1:** Comparisons of remitted versus non-remitted patients according to RSWG-cr (12), *N* = 649

	***N***	**Remitted patients (*n* = 356)**	**Non-remitted patients (*n* = 293)**	***p***
**PANSS-8 sum score (*M*, *SD*)**	649	10.3, 2.8	21.4, 7.0	** < 0.001**^**1**^
**Age, in years (*M*, *SD*)**	649	50.9, 13.8	51.1, 13.9	0.75^2^
**Sex**	649			
Female	290	167 (46.9%)	123 (42.0%)	0.21^3^
Male	359	189 (53.1%)	170 (58.0%)	
**Year of diagnosis**	643			
2009 or earlier	472	256 (72.5%)	216 (74.5%)	0.58^3^
2010 or later	171	97 (27.5%)	74 (25.5%)	
**Country of birth**	647			
Sweden	409	217 (61.3%)	192 (65.5%)	0.27^3^
Outside Sweden	238	137 (38.7%)	101 (34.5%)	
**Level of education**	601			
Completed high school	419	238 (71.9%)	181 (67.0%)	0.20^3^
Not completed high school	182	93 (28.1%)	89 (33.0%)	
**Living situation**	645			
Independent living	343	222 (62.9%)	121 (41.4%)	** < 0.001**^**3**^
Assisted living	302	131 (37.1%)	171 (58.6%)	
**Type of income**	592			
Personal income	184	124 (38.2%)	60 (22.5%)	** < 0.001**^**3**^
Social benefits	408	201 (61.8%)	207 (77.5%)	
**BMI (*M*, *SD*)**	649	29.5, 6.0	28.6, 6.3	0.84^2^
**Obesity**	649	148 (41.6%)	104 (35.5%)	0.11^3^
**Somatic comorbidity (pooled)**	649	102 (28.7%)	89 (30.4%)	0.63^3^
Diabetes	649	48 (13.5%)	50 (17.1%)	0.20^3^
Cardiovascular disease	649	53 (14.9%)	41 (14.0%)	0.75^3^
Thyroid disease	649	23 (6.5%)	14 (4.8%)	0.36^3^
Kidney disease	649	5 (1.4%)	3 (1.0%)	0.66^3^
Chronic obstructive pulmonary disease	649	12 (3.4%)	11 (3.8%)	0.79^3^
Cancer	649	5 (1.4%)	8 (2.7%)	0.23^3^
**Smoker?**	649			
Yes	152	65 (18.3%)	87 (29.7%)	** < 0.001**^**3**^
No	497	291 (81.7%)	206 (70.3%)	
**Antipsychotic medication?**	647			
Yes	592	314 (88.5%)	278 (95.2%)	**0.002**^**3**^
No	55	41 (11.5%)	14 (4.8%)	
**Health interview registered within the past year?**	649			
Yes	432	253 (71.1%)	179 (61.1%)	**0.007**^**3**^
No	217	103 (28.9%)	114 (38.9%)	
**Physical examination registered in the past year?**	649			
Yes	411	243 (68.3%)	168 (57.3%)	**0.004**^**3**^
No	238	113 (31.7%)	125 (42.7%)	

Bold *p* values indicate significant differences at the .05 level

^1^ Mann–Whitney *U* test

^2^ Independent samples *t* test

^3^ Chi-square test

Cronbach’s alpha for the PANSS-8 was 0.86. The Mean PANSS-8 sum score was 10.3 (*SD* = 2.8) in the remitted group and 21.4 (*SD* = 7.0) in the non-remitted group (*p* < 0.001). The groups did not differ significantly in terms of age (*p* = 0.75), sex (*p* = 0.21), place of birth (*p* = 0.27), level of education (*p* = 0.20), or duration of diagnosis (*p* = 0.58).

Living situation differed significantly between the groups, with 63% of patients in remission living independently compared with 41% of non-remitted patients (*p* < 0.001). Type of income also differed significantly, with 38% of remitted patients reporting personal income compared with 22% of unremitted patients (*p* < 0.001).

Regarding somatic comorbidity and risk factors, there were no significant between-group differences in mean BMI (*p* = 0.84), obesity (*p* = 0.11), or general somatic comorbidity (*p* = 0.63; separate chi-square tests for each individual comorbidity were all non-significant with *p* > 0.2). However, smoking was significantly less prevalent among remitted patients – 18% of those were smokers, compared with 30% of non-remitted patients (p < 0.001).

Patients in remission were also less likely than non-remitted patients to be currently treated with antipsychotic medication (88% vs. 95%; *p* = 0.002).

A health interview was registered in the previous year for 71% of remitted patients, compared with 61% of non-remitted patients (*p* = 0.007). Similarly, a physical examination was registered for 68% of remitted patients, compared with 57% of non-remitted patients (*p* = 0.004).

### Objective 2: Independent predictors of remission

To quantify each variable’s independent contribution in predicting remission status, a binary logistic regression analysis was performed with remission status (remission vs. non-remission) as the dependent variable. Bivariate association tests and multicollinearity diagnostics performed before the analysis showed that excluding potential variables of interest was unnecessary. Thus, all 13 were entered into the regression model: age, sex, year of diagnosis, country of birth, level of education, living situation, type of income, obesity, somatic comorbidity, smoking status, antipsychotic medication status, health interview in the past year, and physical examination in the past year. After 110 cases with missing values were excluded, 539 patients were included in the model.

Five variables—living situation, type of income, obesity, antipsychotic medication status, and physical examination in the past year—emerged as statistically significant predictors of remission (*p* < 0.05). Patients living independently had an increased likelihood of being in remission (OR = 1.98, CI: 1.35–2.91), along with those earning their own income (OR = 1.89, CI: 1.25–2.85). Obesity (OR = 1.61, CI: 1.09–2.37) and physical examination in the past year (OR = 1.56, CI: 1.03–2.37) were also linked with a higher likelihood of remission. Patients treated with antipsychotic medication, however, were less likely to be in remission (OR = 0.41, CI: 0.21–0.83). Smoking tended to predict a lower likelihood of remission in the model but was not significant when other factors were controlled for (OR = 0.69, CI: 0.44–1.06, *p* = 0.093).

## Discussion

The PANSS-8 structured according to the RSWG’s 3D model showed the best model fit indices, all of which were acceptable except for a somewhat high NC. However, chi-square values are also sensitive to large samples, usually defined as *n* > 400 [46], and our study included 1,744 observations. Because we evaluated a brief instrument with only eight items, meaning few degrees of freedom, the NC-based approach may have not controlled for the large sample’s effect [54, 55]. Indeed, because chi-square is sensitive to sample size and NC is sensitive to model complexity, the chi-square test may not always be suitable [56].

The EFA suggested a two-factor solution in which item G5 (mannerisms and posturing) within the RSWG-cr’s dimension of disorganization, showed no preference for either factor. Another study has also shown the item’s weak prediction of remission status given the few patients who scored high on it [15].

The PANSS-8 showed good internal reliability in both samples, though the sample for Objective 2 was smaller than the one for Objective 1 (*N* = 649 vs. *N* = 1,744) and scored significantly less on the scale. That finding demonstrates the instrument’s stable internal reliability regardless of patients’ scores. Because alpha values tend to be higher for scales with more items, the good alpha values for the brief 8-item scale underscore its robustness.

In our study, 55% of patients were in remission, as consistent with previously found remission frequencies based on RSWG-cr [57]. Although we could not apply the RSWG’s time criterion, neither have most other studies on remission [58]. One review revealed lower remission frequencies at follow-up in studies including the time criterion than in ones using severity criteria only, albeit the difference was not dramatic (44% vs. 56%) [57].

We examined the RSWG-cr’s clinical validity by identifying associations between remission status and various demographic and clinical variables. Between-group comparisons showed that most remitted patients were living independently and had personal income compared with non-remitted patients. That unsurprising finding may indicate a relationship in either direction. Patients who are initially less well-adjusted (e.g., lack employment prior to first-episode psychosis) are known to achieve worse symptomatic outcomes [59], but remission itself is also conceptualized as a step toward recovery that includes work and social functioning [12]. Although we could not assess the direction of causality in our study, only modifiable socioeconomic variables showed that association with remission, the non-modifiable background data of country of birth and level of education—the latter being unlikely to change given our sample’s relatively high mean age and the use of “Completed high school” as a cutoff—did not differ between remitted and non-remitted patients. That dynamic indicates that our results may indeed reflect remission’s effect on function. Our results also echo past findings of positive associations between remission and two identified indicators of recovery [31, 60]: employment [24, 25] and living independently [25].

The proportion of health interviews and physical examinations conducted was higher among the remitted patients than the non-remitted ones. That finding may reflect remitted patients’ increased motivation and capacity to attend and participate in such procedures, or that patients in more frequent contact with health care services may initially receive earlier intervention and consequently be more likely to achieve remission. After all, the longer that psychosis goes untreated, the worse the outcomes in schizophrenia [36].

Current treatment with antipsychotic medication, though common among remitted patients (88%), was more prevalent among non-remitted ones (95%), which may appear surprising given the well-established benefits of maintenance treatment with antipsychotics in cases of schizophrenia [61]. However, we did not assess adherence to treatment, nor was treatment with other medications (e.g., mood stabilizers) registered. The observed difference may reflect the discontinuation of antipsychotic treatment in less symptomatic patients. Although long-term maintenance treatment with antipsychotics in schizophrenia is recommended by medical guidelines in Sweden [29, 62], some patients may arguably be able to maintain good outcomes without such treatment [63, 64]. Another potential explanation could be different distributions of diagnoses. Although the year of the psychotic disorder’s diagnosis was registered nearly all patients in our smaller sample—data were missing for only 6 of 649 patients—the exact diagnoses were not. Thus, patients with less severe diagnoses (e.g., delusional disorder or schizophreniform disorder vs. schizophrenia), who are more likely to have discontinued antipsychotic treatment [64], may have been disproportionately represented in the remitted sample.

Obesity emerged as a significant independent predictor of remission in logistic regression analysis. Repeating the logistic regression analysis with BMI as a continuous variable instead of obesity as a predictor yielded similar results (data not shown). That finding may be surprising given the well-known association between schizophrenia and obesity [65]. However, high rates of obesity among patients with schizophrenia can be partly explained by antipsychotic treatment, in which the antipsychotics most efficient in reducing symptoms (e.g., clozapine and olanzapine) also tend to be associated with more severe metabolic side effects [37]. The specific antipsychotic medication used, though not registered in our data, may have differed between groups. Thus, patients who were treated with more metabolically neutral compounds (e.g., aripiprazole) may have been less prone to obesity and to achieve symptomatic remission. A direct positive relationship between treatment-induced weight gain and symptom remission, independent of treatment dose and duration, has been observed, even for less obesogenic antipsychotics [66].

### Strengths and limitations

Our naturalistic study used a large, heterogeneous sample including patients up to 93 years old, which is an advantage because older adults, who contribute largely to the general population’s health burden, are often excluded from clinical trials, which limits their ability to benefit from such research [67]. Strict inclusion criteria regarding factors such as age and comorbidity, with the primary intention of increasing the statistical power and simplicity of trials, may limit the generalizability of our results to the wider clinical population. However, we used data from annual health checkups offered to all patients admitted in psychosis clinics in Gothenburg, Sweden, which afforded the sample good clinical representativeness. For example, unlike in other studies, we did not exclude patients with comorbidities (e.g., addiction, autism spectrum disorder, and physical disability) [15, 24].

Data collected during the checkups were also sent to the Swedish National Psychosis Registry, which, though suffering from low coverage, contains data from 7,000–8,000 new registrations of patients with psychosis each year [68]. Comparing our sample with the larger national sample elucidates its representativeness. Our sample was fairly similar to the national sample, in which 46% of patients were in remission per the RSWG-cr, 61% lived independently, and 42% were obese, compared with 55%, 53%, and 39% in our study, respectively. Smoking status was an exception, and the considerably higher prevalence of smoking in the national registry (43 vs. 23%) raises suspicions that patients with missing information about smoking status may have been miscoded as nonsmokers in our data.

Although health checkups are offered to all patients admitted to Gothenburg’s outpatient psychosis clinics, our findings represent only a subset of them. Reasons for not receiving a checkup may include patient-based (e.g., lack of insight, lack of motivation, and planning difficulties) and organizational factors (e.g., structural reorganizations, time constraints, and difficulty rescheduling missed appointments), which could have induced selection bias in favor of relatively healthy patients [68]. Many of the PANSS-8’s items showed pronounced floor effects, thereby suggesting mild symptom severity, if any. However, the percentage of patients in remission was similar to that in other studies.

Some variables that would have been useful in interpreting our analyses’ results were not registered in our study. First, specific psychotic diagnoses were not coded. Although most patients in the registry have schizophrenia, other types of psychotic disorders are also represented [69] buy may differ in their clinical presentation. Specific information about pharmaceutical treatments, including antipsychotic compounds, doses, polypharmacy, and non-antipsychotic treatment, could also help to draw conclusions from the data. Data on non-pharmacologic treatment, including psychotherapy, were also unavailable.

The major limitation stems from our study’s cross-sectional design, which required omitting the RSWG-cr’s time component despite the importance of duration for conceptualizing remission [58]. Analyses were also limited to measures of correlation that do not allow assessing causality. However, confounders were somewhat controlled for via logistic regression. Future longitudinal observations of similar naturalistic samples would be valuable given the possibility of using the full RSWG-cr including the time-related criteria and observing time-affected relationships between remission and associated variables while maintaining the sample’s heterogeneous, clinically representative nature.

## Conclusions

Our study contributes to the literature by evaluating the PANSS-8 using an unusually heterogeneous, clinically representative sample of outpatients with psychotic disorders. Of all the models evaluated, the model with three dimensions—psychoticism, disorganization, and negative symptoms—displayed the best model fit along with good internal reliability. Per the RSWG-cr, being employed and living independently were the strongest independent statistical predictors of remission. As important clinical components of functional recovery, those factors undergird the RSWG-cr’s clinical relevance.

## Supplementary Information

**Additional file 1.**

**Additional file 2.**

**Additional file 3.**

**Additional file 4.**

## Data Availability

The data used in the study will not be shared publicly, because doing so risks compromising patients’ privacy. However, the data can be made available upon legitimate request made to the corresponding author, KS.

## Abbreviations

CFA: Confirmatory factor analysis
EFA: Exploratory factor analysis
NC: Normed chi-square
PANSS: Positive and negative syndrome scale
RSWG: Remission in schizophrenia working group
RSWG-cr: Remission in schizophrenia working group’s criteria

## References

[1] American Psychiatric Association (2013). Diagnostic and statistical manual of mental disorders: DSM-5.

[2] Charlson FJ, Ferrari AJ, Santomauro DF, Diminic S, Stockings E, Scott JG (2018). Global Epidemiology and Burden of Schizophrenia: Findings From the Global Burden of Disease Study 2016. Schizophr Bull.

[3] Vancampfort D, Wampers M, Mitchell AJ, Correll CU, De Herdt A, Probst M (2013). A meta-analysis of cardio-metabolic abnormalities in drug naïve, first-episode and multi-episode patients with schizophrenia versus general population controls. World Psychiatry.

[4] Crump C, Winkleby MA, Sundquist K, Sundquist J (2013). Comorbidities and mortality in persons with schizophrenia: a Swedish national cohort study. Am J Psychiatry.

[5] Lieberman JA, First MB (2018). Psychotic Disorders. N Engl J Med.

[6] Gaebel W, Zielasek J (2015). Focus on psychosis. Dialogues Clin Neurosci.

[7] Bottlender R, Strauss A, Möller HJ (2013). Association between psychopathology and problems of psychosocial functioning in the long-term outcome of patients diagnosed with schizophrenic, schizoaffective and affective disorders. Eur Arch Psychiatry Clin Neurosci.

[8] Emsley R, Chiliza B, Asmal L, Lehloenya K (2011). The concepts of remission and recovery in schizophrenia. Curr Opin Psychiatry..

[9] Frese FJ, Knight EL, Saks E (2009). Recovery from schizophrenia: with views of psychiatrists, psychologists, and others diagnosed with this disorder. Schizophr Bull.

[10] van Os J, Burns T, Cavallaro R, Leucht S, Peuskens J, Helldin L (2006). Standardized remission criteria in schizophrenia. Acta Psychiatr Scand.

[11] Leucht S, Lasser R (2006). The concepts of remission and recovery in schizophrenia. Pharmacopsychiatry.

[12] Andreasen NC, Carpenter WT, Kane JM, Lasser RA, Marder SR, Weinberger DR (2005). Remission in Schizophrenia: Proposed Criteria and Rationale for Consensus. AJP.

[13] Kay SR, Fiszbein A, Opler LA (1987). The positive and negative syndrome scale (PANSS) for schizophrenia. Schizophr Bull.

[14] Gorwood P, Peuskens J (2012). Setting new standards in schizophrenia outcomes: symptomatic remission 3 years before versus after the Andreasen criteria. Eur Psychiatry.

[15] Johansson M, Hjärthag F, Helldin L (2018). What could be learned from a decade with standardized remission criteria in schizophrenia spectrum disorders: An exploratory follow-up study. Schizophr Res.

[16] Catalan A, Richter A, Salazar de Pablo G, Vaquerizo-Serrano J, Mancebo G, Pedruzo B (2021). Proportion and predictors of remission and recovery in first-episode psychosis: Systematic review and meta-analysis. Eur Psychiatry..

[17] Bınbay T, Ergül C, van Os J (2021). Symptomatic Remission Along the Clinical Psychosis Spectrum: A Historical and Conceptual Review. Noro Psikiyatr Ars..

[18] Lahera G, Gálvez JL, Sánchez P, Martínez-Roig M, Pérez-Fuster JV, García-Portilla P (2018). Functional recovery in patients with schizophrenia: recommendations from a panel of experts. BMC Psychiatry.

[19] Lahera G, Pérez-Fuster V, Gálvez JL, Martínez M, Sánchez P, Roca M (2016). Is it possible to achieve functional recovery in schizophrenia? A qualitative and quantitative analysis of psychiatrist’s opinion. Actas Esp Psiquiatr.

[20] Huxley P, Krayer A, Poole R, Prendergast L, Aryal S, Warner R (2021). Schizophrenia outcomes in the 21st century: A systematic review. Brain Behav.

[21] Bouwmans C, de Sonneville C, Mulder CL, Hakkaart-van RL (2015). Employment and the associated impact on quality of life in people diagnosed with schizophrenia. Neuropsychiatr Dis Treat.

[22] Chang WC, Chan TCW, Chen ESM, Hui CLM, Wong GHY, Chan SKW (2013). The concurrent and predictive validity of symptomatic remission criteria in first-episode schizophrenia. Schizophr Res.

[23] Heering HD, Janssens M, Boyette LL, van Haren NEM, G.R.O.U.P investigators (2015). Remission criteria and functional outcome in patients with schizophrenia, a longitudinal study. Aust N Z J Psychiatry..

[24] Wang SP, Wang JD, Chang JH, Wu BJ, Wang TJ, Sun HJ (2020). Symptomatic remission affects employment outcomes in schizophrenia patients. BMC Psychiatry.

[25] Helldin L, Kane JM, Karilampi U, Norlander T, Archer T (2007). Remission in prognosis of functional outcome: A new dimension in the treatment of patients with psychotic disorders. Schizophr Res.

[26] Oorschot M, Lataster T, Thewissen V, Lardinois M, van Os J, Delespaul P a. EG (2012). Symptomatic remission in psychosis and real-life functioning. Br J Psychiatry..

[27] Carpiniello B, Pinna F, Manchia M, Tusconi M, Cavallaro R, Bosia M (2022). Sustained symptomatic remission in schizophrenia: Course and predictors from a two-year prospective study. Schizophr Res.

[28] Pinna F, Tusconi M, Bosia M, Cavallaro R, Carpiniello B, the Cagliari Recovery Group Study (2013). Criteria for symptom remission revisited: a study of patients affected by schizophrenia and schizoaffective disorders. BMC Psychiatry..

[29] Svenska Psykiatriska Föreningen [Swedish Psychiatric Association]. Schizofreni - kliniska riktlinjer för utredning och behandling [Schizophrenia - clinical guidelines for investigation and treatment]. 2nd ed. Laurell BW, editor. Stockholm: Gothia Förlag; 2009.

[30] Holmberg C, Gremyr A, Torgerson J, Mehlig K (2021). Clinical validity of the 12-item WHODAS-2.0 in a naturalistic sample of outpatients with psychotic disorders. BMC Psychiatry..

[31] Liberman R, Kopelowicz A, Ventura J, Gutkind D (2002). Operational criteria and factors related to recovery from schizophrenia. International Review of Psychiatry - Int Rev Psychiatr.

[32] Cantor-Graae E, Selten JP (2005). Schizophrenia and Migration: A Meta-Analysis and Review. AJP.

[33] Marwaha S, Johnson S (2004). Schizophrenia and employment - a review. Soc Psychiatry Psychiatr Epidemiol.

[34] Quigley H, MacCabe JH. The relationship between nicotine and psychosis. Therapeutic Advances in Psychopharmacology [Internet]. 2019;9. Available from: https://journals.sagepub.com/doi/10.1177/2045125319859969. 10.1177/2045125319859969 PMC6604123 31308936

[35] Beary M, Hodgson R, Wildgust HJ (2012). A critical review of major mortality risk factors for all-cause mortality in first-episode schizophrenia: clinical and research implications. J Psychopharmacol.

[36] Emsley R, Chiliza B, Schoeman R (2008). Predictors of long-term outcome in schizophrenia. Curr Opin Psychiatry.

[37] Pillinger T, McCutcheon RA, Vano L, Mizuno Y, Arumuham A, Hindley G (2020). Comparative effects of 18 antipsychotics on metabolic function in patients with schizophrenia, predictors of metabolic dysregulation, and association with psychopathology: a systematic review and network meta-analysis. Lancet Psychiatry.

[38] Lawrence D, Kisely S (2010). Inequalities in healthcare provision for people with severe mental illness. J Psychopharmacol.

[39] Świtaj P, Anczewska M, Chrostek A, Sabariego C, Cieza A, Bickenbach J (2012). Disability and schizophrenia: a systematic review of experienced psychosocial difficulties. BMC Psychiatry.

[40] Kumari V, Postma P (2005). Nicotine use in schizophrenia: the self medication hypotheses. Neurosci Biobehav Rev.

[41] Barnes M, Lawford BR, Burton SC, Heslop KR, Noble EP, Hausdorf K (2006). Smoking and schizophrenia: is symptom profile related to smoking and which antipsychotic medication is of benefit in reducing cigarette use?. Aust N Z J Psychiatry.

[42] Socialstyrelsen. Nationella riktlinjer för vård och stöd vid schizofreni och schizofreniliknande tillstånd - Stöd för styrning och ledning. www.socialstyrelsen.se; 2018.

[43] Jackson DL, Gillaspy JA, Purc-Stephenson R (2009). Reporting practices in confirmatory factor analysis: An overview and some recommendations. Psychol Methods.

[44] Kim HY (2013). Statistical notes for clinical researchers: assessing normal distribution (2) using skewness and kurtosis. Restorative Dentistry & Endodontics.

[45] Hu L, Bentler PM (1999). Cutoff criteria for fit indexes in covariance structure analysis: Conventional criteria versus new alternatives. Struct Equ Modeling.

[46] Stone BM (2021). The Ethical Use of Fit Indices in Structural Equation Modeling: Recommendations for Psychologists. Front Psychol.

[47] Schumacker RE, Lomax RG (2010). A beginner’s guide to structural equation modeling.

[48] Portney LG, Watkins MP. Foundations of clinical research : applications to practice. 3rd ed. Upper Saddle River, N.J: Pearson / Prentice Hall. 2008:125.

[49] Lance CE, Butts MM, Michels LC (2006). The Sources of Four Commonly Reported Cutoff Criteria: What Did They Really Say?. Organ Res Methods.

[50] Eisinga R, te Grotenhuis M, Pelzer B (2013). The reliability of a two-item scale: Pearson, Cronbach, or Spearman-Brown?. Int J Public Health.

[51] Yong AG, Pearce S (2013). A Beginner’s Guide to Factor Analysis: Focusing on Exploratory Factor Analysis. TQMP.

[52] Kim JH (2019). Multicollinearity and misleading statistical results. Korean J Anesthesiol.

[53] Ward RC, Axon RN, Gebregziabher M (2020). Approaches for missing covariate data in logistic regression with MNAR sensitivity analyses. Biom J.

[54] Schermelleh-Engel K, Moosbrugger H, Müller H (2003). Evaluating the Fit of Structural Equation Models: Tests of Significance and Descriptive Goodness-of-Fit Measures. Methods Psychol Res Online.

[55] Alavi M, Visentin DC, Thapa DK, Hunt GE, Watson R, Cleary M (2020). Chi-square for model fit in confirmatory factor analysis. J Adv Nurs.

[56] West SG, Taylor AB, Wu W. Model fit and model selection in structural equation modeling. Handbook of structural equation modeling. The Guildford Press; 2012;1:209–31.

[57] Lambert M, Karow A, Leucht S, Schimmelmann BG, Naber D (2010). Remission in schizophrenia: validity, frequency, predictors, and patients’ perspective 5 years later. Dialogues Clin Neurosci.

[58] AlAqeel B, Margolese HC (2012). Remission in Schizophrenia: Critical and Systematic Review: Harvard Review of Psychiatry.

[59] Petersen L, Thorup A, Øqhlenschlaeger J, Christensen TØ, Jeppesen P, Krarup G (2008). Predictors of remission and recovery in a first-episode schizophrenia spectrum disorder sample: 2-year follow-up of the OPUS trial. Can J Psychiatry.

[60] Kane JM, Leucht S, Carpenter D, Docherty JP, Expert Consensus Panel for Optimizing Pharmacologic Treatment of Psychotic Disorders (2003). The expert consensus guideline series. Optimizing pharmacologic treatment of psychotic disorders. Introduction: methods, commentary, and summary. J Clin Psychiatry..

[61] Ceraso A, Lin JJ, Schneider-Thoma J, Siafis S, Tardy M, Komossa K, et al. Maintenance treatment with antipsychotic drugs for schizophrenia. Cochrane Database Syst Rev. 2020;8:CD008016. 10.1002/14651858.CD008016.pub3 PMC9702459 32840872

[62] Läkemedelsbehandling vid schizofreni - behandlingsrekommendation [Pharmacological treatment in schizophrenia - treatment recommendation]: Information från Läkemedelsverket [Information from the Swedish Medical Products Agency]. 2013;24(5):15–27.

[63] Murray RM, Quattrone D, Natesan S, van Os J, Nordentoft M, Howes O (2016). Should psychiatrists be more cautious about the long-term prophylactic use of antipsychotics?. Br J Psychiatry.

[64] Moilanen J, Haapea M, Miettunen J, Jääskeläinen E, Veijola J, Isohanni M (2013). Characteristics of subjects with schizophrenia spectrum disorder with and without antipsychotic medication - a 10-year follow-up of the Northern Finland 1966 Birth Cohort study. Eur Psychiatry.

[65] Manu P, Dima L, Shulman M, Vancampfort D, De Hert M, Correll CU (2015). Weight gain and obesity in schizophrenia: epidemiology, pathobiology, and management. Acta Psychiatr Scand.

[66] Luckhoff H, Phahladira L, Scheffler F, Asmal L, du Plessis S, Chiliza B (2019). Weight gain and metabolic change as predictors of symptom improvement in first-episode schizophrenia spectrum disorder patients treated over 12 months. Schizophr Res.

[67] Herrera AP, Snipes SA, King DW, Torres-Vigil I, Goldberg DS, Weinberg AD (2010). Disparate Inclusion of Older Adults in Clinical Trials: Priorities and Opportunities for Policy and Practice Change. Am J Public Health.

[68] PsykosR (2019). Nationellt Kvalitetsregister för psykossjukdomar..

[69] Holmberg C, Gremyr A, Karlsson V, Asztély K (2022). Digitally excluded in a highly digitalized country: An investigation of Swedish outpatients with psychotic disorders and functional impairments. European Journal of Psychiatry.

