# Supplementary material for: Psychometric and clinical evaluation of schizophrenia remission criteria in outpatients with psychotic disorders
Source: BMC Psychiatry. 2023 Mar 28;23:207. doi: 10.1186/s12888-023-04701-3 (PMC10052840; doi:10.1186/s12888-023-04701-3)
Supplement: Supplementary file 1 — Additional file 1. [file 12888_2023_4701_MOESM1_ESM.pdf]

Annual follow-up form (transl. from Swedish)

Patient personal identity number:________________________________

Name:____

Case manager:____

Information date:_________________________(YYYY-MM-DD)

*

**Questions beginning with an asterisk (*) are filled in at the first annual follow-up. Because these refer to data that do not change over time, they do not need to be filled in again if an annual follow-up has been carried out during the previous year.*

About quality registers

*Is the patient informed about national quality registers? Circle one option.

Annual follow-up is carried out for all patients. Data can be forwarded to PsykosR and Kvalitetsstjärnan [relevant national quality registers] unless the patient actively refuses participation. If the patient has already been included in registers, e.g., during the previous year, use "Yes, and does not refuse participation".

○ **Yes, and does not refuse participation**

○ **Yes, and refuses participation in PsykosR**

○ **Yes, and refuses participation in any national quality register**

○ **No, the patient is not informed**

Patient situation and background

*What year did the patient fall ill? Circle one option.

Refers to diagnosis resulting in contact with Psychiatry Psychosis, regardless of whether this is a psychotic diagnosis.

.

○ **2009 or earlier** ○ **2012** ○ **2015**

○ **2010** ○ **2013** ○ **2016**

○ **2011** ○ **2014** ○ **2017**

○ **2018** ○ **2019** ○ **2020**

*Where was the patient born? Circle one option.
Refers to ”Birth area” as is it used in the local quality register.

○ **Sweden**

○ **Scandinavia (except Sweden)**

○ **Europe (except Sweden and Scandinavia)**

○ **North America**

○ **South America**

○ **Asia**

○ **Africa**

○ **Oceania**

*Has the patient experienced involuntary treatment? If “yes”, when? Circle one option.
Refers to involuntary psychiatric treatment in general regardless of where.

○ **No (the patient has not received involuntary treatment)**

○ **Yes, before 2012**

○ **Yes, 2012 or later**

*If ”Yes,…”, has the patient evaluated the time of involuntary treatment (according to ISP)? Circle one option.

○ **Yes**

○ **Not yet**

○ **Offered to the patient but declined**

How does the patient live? Circle one option.
Homeless refers to a person who lacks owned or rented housing and who does not live in a steady live-in or subletting arrangement, and who is dependent on temporary housing options or sleeps rough.

○ **Ordinary housing without assistance (by social services or equivalent)**

○ **Ordinary housing with assistance (by social services or equivalent)**

○ **Permanent special housing (acc. to SoL/LSS) with staff assistance part of the day**

○ **Permanent special housing (acc. to SoL/LSS) with 24-hour staff assistance**

○ **Family home**

○ **Housing with social contract**

○ **Homeless** *(see definition below the question)*

○ **Data missing**

What is the household composition?

Circle one or more options.

○ **Living alone**

○ **Household shared with wife/husband/partner**

○ **Household shared with parent/parents**

○ **Household shared with other adults (including own children aged 18 or older)**

○ **Hosehold shared with children below 18**

○ **Data missing**

Are there underage children who are affected by the patient's illness?

Information about underage children is noted in the digital journal system

"No" – if there are no children

"Yes", birth year, and custody (Shared, Own or No custody) for each child.

Birth year (YYYY)	Custody (Shared, Own or No custody)

What is the patient's highest level of education?
Circle the option that fits best.

○ **Not completed pre-high school education**

○ **Completed pre-high school education**

○ **Completed high school education**

○ **Completed further education (at least 2 years)**

○ **Completed further education (at least 3 years)**

What is the patient's main occupation the last 12 months?
Circle one or more options.

○ **Employment/education at the open market more that 50 percent**

○ **Employment/education at the open market 50 percent or less**

○ **Unemployed/labour market measure**

○ **Protected occupation more than 50 percent**

○ **Protected occupation 50 percent or less**

○ **Tailored education (goal further education/work)**

○ **Occupational rehabilitation (incl. under investigation to clarify functional level)**

○ **Employment corresponding to SOL's definition (day centers, user clubs, etc.)**

○ **No occupation**

○ **Data missing**

What is the patient's main source of income the last 12 months?

About persons who are on sick leave, who are not entitled to sickness benefits and therefore receive compensation from social services, the option "Financial assistance according to SOL or equivalent" is indicated. Own income refers to paid work, self-employed, student funds, old-age pension/guaranteed pension. Several answer options can be entered - a maximum of two may be chosen.

○ **Own income**

○ **Unemployment benefits**

○ **Parental benefits**

○ **Sickness benefits or equivalent**

○ **Financial assistance according to SOL or equivalent**

○ **Supported by a relative**

○ **Own capital**

○ **Other**

○ **Data missing**

Risk conditions and antipsychotic medications

Has the patient ever attempted suicide?
Attempted suicide refers to life-threatening or seemingly life-threatening behavior, with the intention of putting one's life at risk or giving the impression of such an intention, but which does not lead to death (e.g., serious tablet poisoning, drowning, shooting, hanging/strangulation)

○ **No**

○ **Yes**

If yes; state when suicide attempts occurred (if they occurred later than 2017-01-01 and have not yet been documented in the follow-up database)

Date:

Circle current risk conditions and somatic diagnoses
If there are none of the risk conditions and diagnoses below, mark "Not applicable".

○ **Diabetes**

○ **Cancer**

○ **Thyroid disease**

○ **Breastfeeding**

○ **COPD**

○ **Cardiovascular disease**

○ **Kidney disease**

○ **Pregnancy**

○ **Smoking** (daily smoker)

○ **Not applicable**

Is the patient being treated with antipsychotic medications? ○ **Yes** ○ **No**

Now two pages follow. The first contains a remission assessment (PANSS-8) which is done by you as CM/clinician if the patient has a psychotic illness. The second is a patient assessment of functional Level (WHODAS). The patient assessment is done by people with all different types of diagnoses.

Remission assessment (PANSS-8) – Clinician assessment of psychotic disease

P1 Delusions
One answer option can be chosen
○ **1 Absent**
○ **2 Minimal**
○ **3 Mild**
○ **4 Moderate**
○ **5 Moderate severe**
○ **6 Severe**
○ **7 Extreme**
○ **8 Data missing**

P2 Conceptual disorganization
One answer option can be chosen
○ **1 Absent**
○ **2 Minimal**
○ **3 Mild**
○ **4 Moderate**
○ **5 Moderate severe**
○ **6 Severe**
○ **7 Extreme**
○ **8 Data missing**

P3 Hallucinatory behavior
One answer option can be chosen
○ **1 Absent**
○ **2 Minimal**
○ **3 Mild**
○ **4 Moderate**
○ **5 Moderate severe**
○ **6 Severe**
○ **7 Extreme**
○ **8 Data missing**

N1 Blunted affect
One answer option can be chosen
○ **1 Absent**
○ **2 Minimal**
○ **3 Mild**
○ **4 Moderate**
○ **5 Moderate severe**
○ **6 Severe**
○ **7 Extreme**
○ **8 Data missing**

N4 Passive/apathetic social withdrawal
One answer option can be chosen
○ **1 Absent**
○ **2 Minimal**
○ **3 Mild**
○ **4 Moderate**
○ **5 Moderate severe**
○ **6 Severe**
○ **7 Extreme**
○ **8 Data missing**

N6 Lack of spontaneity and flow of conversation
One answer option can be chosen
○ **1 Absent**
○ **2 Minimal**
○ **3 Mild**
○ **4 Moderate**
○ **5 Moderate severe**
○ **6 Severe**
○ **7 Extreme**
○ **8 Data missing**

A5 Mannerisms and posturing
One answer option can be chosen
○ **1 Absent**
○ **2 Minimal**
○ **3 Mild**
○ **4 Moderate**
○ **5 Moderate severe**
○ **6 Severe**
○ **7 Extreme**
○ **8 Data missing**

A9 Unusual thought content
One answer option can be chosen
○ **1 Absent**
○ **2 Minimal**
○ **3 Mild**
○ **4 Moderate**
○ **5 Moderate severe**
○ **6 Severe**
○ **7 Extreme**
○ **8 Data missing**

This questionnaire asks about difficulties due to health conditions. Health conditions include diseases or illnesses, other health problems that may be short or long lasting, injuries, mental or emotional problems, and problems with alcohol or drugs.

Think back over the past 30 days and answer these questions, thinking about how much difficulty you had doing the following activities. For each question, please circle only one response.

In the past 30 days, how much difficulty did you have due to your health conditions in:

S1	**Standing for long periods such as 30 minutes?**	*None*	*Mild*	*Moderate*	*Severe*	*Extreme or cannot do*
S2	**Taking care of your household responsibilities?**	*None*	*Mild*	*Moderate*	*Severe*	*Extreme or cannot do*
S3	**Learning a new task, for example, learning how to get to a new place?**	*None*	*Mild*	*Moderate*	*Severe*	*Extreme or cannot do*
S4	**How much of a problem did you have joining in community activities (for example, festivities, religious or other activities) in the same way as anyone else can?**	*None*	*Mild*	*Moderate*	*Severe*	*Extreme or cannot do*
S5	**How much have you been emotionally affected by your health problems?**	*None*	*Mild*	*Moderate*	*Severe*	*Extreme or cannot do*
S6	**Concentrating on doing something for ten minutes?**	*None*	*Mild*	*Moderate*	*Severe*	*Extreme or cannot do*
S7	**Walking a long distance such as a kilometre?**	*None*	*Mild*	*Moderate*	*Severe*	*Extreme or cannot do*
S8	**Washing your whole body?**	*None*	*Mild*	*Moderate*	*Severe*	*Extreme or cannot do*
S9	**Getting dressed?**	*None*	*Mild*	*Moderate*	*Severe*	*Extreme or cannot do*
S10	**Dealing with people you do not know?**	*None*	*Mild*	*Moderate*	*Severe*	*Extreme or cannot do*
S11	**Maintaining a friendship?**	*None*	*Mild*	*Moderate*	*Severe*	*Extreme or cannot do*
S12	**Your day-to-day work or studies?**	*None*	*Mild*	*Moderate*	*Severe*	*Extreme or cannot do*

This was the final question. Thank you for your participation!

WHODAS 2.0
WORLD HEALTH ORGANIZATION
DISABILITY ASSESSMENT SCHEDULE 2.0
