# Supplementary material for: Psychometric and clinical evaluation of schizophrenia remission criteria in outpatients with psychotic disorders
Source: BMC Psychiatry. 2023 Mar 28;23:207. doi: 10.1186/s12888-023-04701-3 (PMC10052840; doi:10.1186/s12888-023-04701-3)
Supplement: Supplementary file 2 — Additional file 2. [file 12888_2023_4701_MOESM2_ESM.pdf]

Tests of normality of distributions

Age:

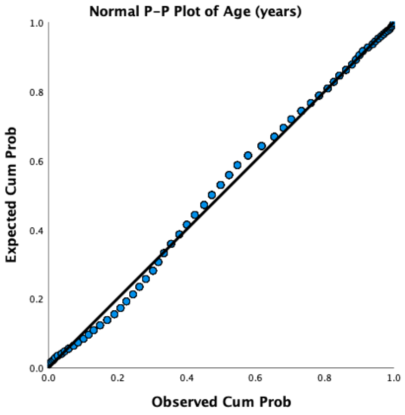

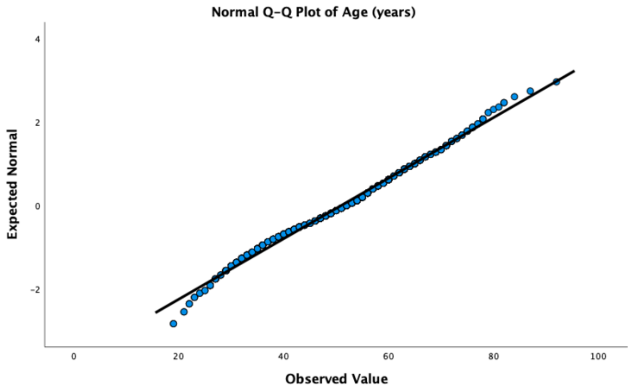

Skewness: -0.03
Kurtosis: -0.56

BMI:

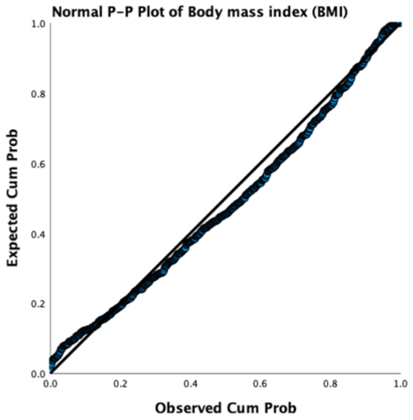

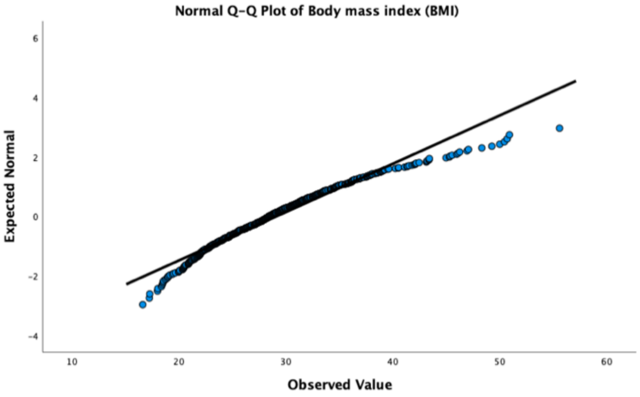

Skewness: 0.85
Kurtosis: 1.12

PANSS-8 sum score:

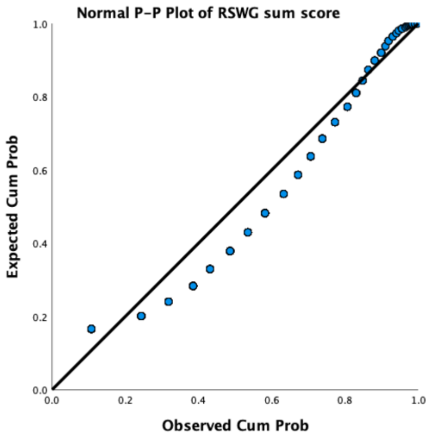

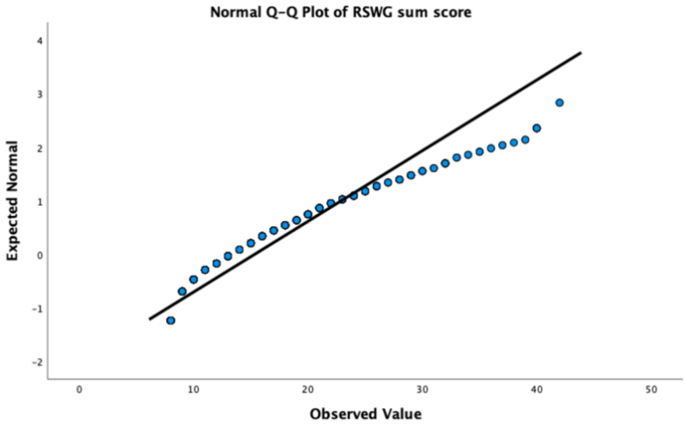

Skewness: 1.25
Kurtosis: 1.18
