# Supplementary material for: Psychometric and clinical evaluation of schizophrenia remission criteria in outpatients with psychotic disorders
Source: BMC Psychiatry. 2023 Mar 28;23:207. doi: 10.1186/s12888-023-04701-3 (PMC10052840; doi:10.1186/s12888-023-04701-3)
Supplement: Supplementary file 3 — Additional file 3. [file 12888_2023_4701_MOESM3_ESM.pdf]

**Item score distributions of the PANSS-8 (N = 1744)**

| Item | Mean (SD[a]) | Median (range) | Floor[b] | Ceiling[c] |
|---|---|---|---|---|
| **P1 Delusions** *(positive symptom, psychoticism)* | 2.37 (1.65) | 2 (1–7) | 47% | 1% |
| **G9 Unusual thought content** *(general symptom, psychoticism)* | 2.00 (1.38) | 1 (1–7) | 56% | 1% |
| **P3 Hallucinatory behaviour** *(positive symptom, psychoticism)* | 1.99 (1.50) | 1 (1–7) | 62% | 0% |
| **P2 Conceptual disorganization** *(positive symptom, disorganization)* | 1.95 (1.33) | 1 (1–7) | 56% | 0% |
| **G5 Mannerisms/Posturing** *(general symptom, disorganization)* | 1.59 (1.03) | 1 (1–7) | 68% | 0% |
| **N1 Blunted affect** *(negative symptom)* | 2.07 (1.29) | 2 (1–7) | 48% | 0% |
| **N4 Social withdrawal** *(negative symptom)* | 2.38 (1.54) | 2 (1–7) | 42% | 1% |
| **N6 Lack of spontaneity** *(negative symptom)* | 2.04 (1.38) | 1 (1–7) | 53% | 0% |
| **Total score** | 16.39 (7.76) | 15 (8–51) | 16% | 0% |

[b] % of lowest possible score
[c] % of highest possible score

The sub-classification of items according to two different models (positive/negative/general symptoms or psychoticism/disorganization/negative symptoms) is indicated in parentheses.
