# Supplementary material for: Psychometric and clinical evaluation of schizophrenia remission criteria in outpatients with psychotic disorders
Source: BMC Psychiatry. 2023 Mar 28;23:207. doi: 10.1186/s12888-023-04701-3 (PMC10052840; doi:10.1186/s12888-023-04701-3)

Factor analyses

Underlined indices do not meet criteria for acceptable fit. Reference values are NC <5.00, CFI >0.90, TLI >0.90, SRMR <0.08, RMSEA <0.10.

Confirmatory factor analysis (CFA) 1:
The first CFA model used the three original PANSS subscales (positive symptoms, general symptoms, and negative symptoms) as latent factors. Fit indices were as follows: $\chi 2$=409.333, df=17 (p < 0.000), NC=24.078, CFI=0.934, TLI=0.892, SRMR=0.063, and RMSEA=0.115 [CI: 0.106–0.125, 90%], indicating poor fit. Furthermore, the factors representing positive and general symptoms had a high degree of overlap (r=0.93).

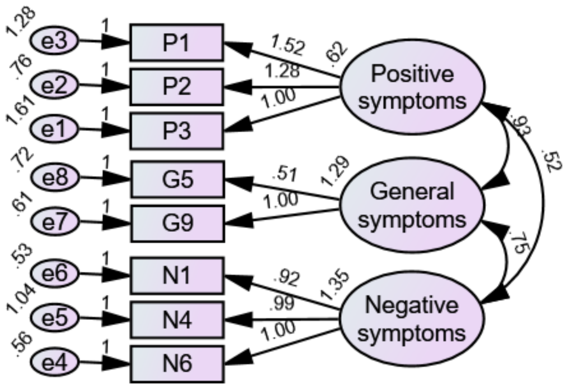

CFA 2:
The second CFA model used the three dimensions of psychoticism, disorganization, and negative symptoms as latent factors. Fit indices: $\chi 2$=234.910, df=17 (p < 0.000), NC=13.818, CFI=0.967, TLI=0.940, SRMR=0.044, and RMSEA=0.086 [CI: 0.076–0.096, 90%]. The model fit was substantially better than that of the first model, but NC (normed chi-square) remained unacceptably high.

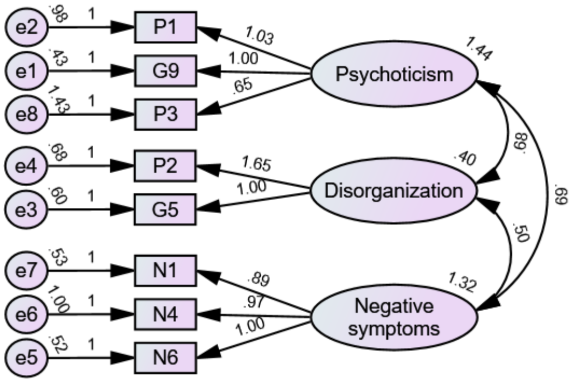

Exploratory factor analysis (EFA):
A principal components analyses was performed. KMO (Kaiser-Meyer-Olkin measure of sampling adequacy) was 0.84, indicating that the sample was suitable for EFA (54).

Below are the initial components (factors) produced by the analysis. Factors with eigenvalues >1, i.e., 1 and 2, were retained.

Factor	Eigenvalue	Percent of variance explained	Cumulative percent of variance explained
1	3.93	49.1	49.1
2	1.34	16.8	65.9
3	.72	9.1	75.0
4	.61	7.7	82.7
5	.43	5.43	88.1
6	.37	4.7	92.8
7	.29	3.7	96.4
8	.29	3.6	100.0

To facilitate interpretation of the data, components were rotated using Varimax rotation. The rotated factor loadings, reflecting the strength of the relationships between each PANSS-8 item and the two factors (possible values ranging between 0 and 1), are reported below.

	Factor 1	Factor 2
P1 (delusions)	.852	.080
P2 (conceptual disorganization)	.716	.347
P3 (hallucinatory behaviour)	.652	.167
N1 (blunted affect)	.184	.858
N4 (social withdrawal)	.213	.796
N6 (lack of spontaneity)	.158	.875
G5 (mannerisms/posturing)	.473	.488
G9 (unusual thought content)	.845	.200

Four items (P1, P2, P3, G9) loaded preferentially on factor 1, labelled "positive symptoms and unusual thought content" below. Three items (N1, N4, N6) loaded preferentially on factor 2, labelled "negative symptoms". The remaining factor G5 had substantial cross-loadings (Factor 1: 0.473, Factor 2: 0.488). This was also the case when performing a Promax rotation (Factor 1: 0.387, Factor 2: 0.405).

CFA 3:

A final CFA was performed on a model containing the two factors extracted from the EFA. Item G5 was removed, leaving 7 items. The following fit indices were obtained: χ^2= 224.341, df=13 ($p < 0.000$), NC=17.257, CFI=0.960, TLI=0.935, SRMR=0.048, and RMSEA=0.097 [CI: 0.086–0.108, 90%]. Thus, NC (normed chi-square) was too high, indicating non-satisfactory fit.

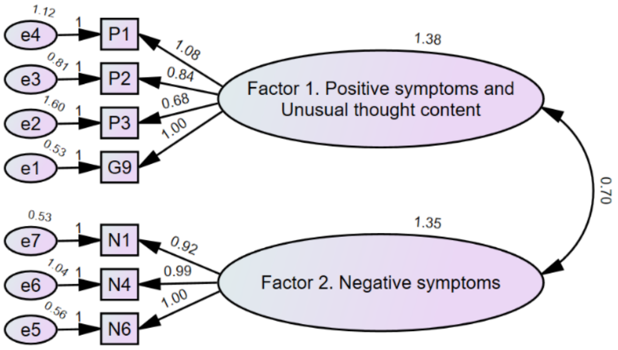

Supplement: Supplementary file 4 — Additional file 4. [file 12888_2023_4701_MOESM4_ESM.pdf]
